# An elaborated feeding cycle model for reductions in vectorial capacity of night-biting mosquitoes by insecticide-treated nets

**DOI:** 10.1186/1475-2875-6-10

**Published:** 2007-01-25

**Authors:** Arnaud Le Menach, Shannon Takala, F Ellis McKenzie, Andre Perisse, Anthony Harris, Antoine Flahault, David L Smith

**Affiliations:** 1 Universite Pierre et Marie Curie-Paris 6, UMR S 707, Paris, F-75012; INSERM, UMR-S 707, Paris, F-75012, France; 2 Center for Vaccine Development, University of Maryland Baltimore, 685 W. Baltimore Street, Baltimore, MD 21201, USA; 3 Division of Epidemiology and Population Studies Fogarty International Center, NIH, Bethesda, MD 20892, USA; 4 Department of Epidemiology and Preventive Medicine, University of Maryland Baltimore, 100 N. Greene Street, Baltimore, MD 21201, USA

## Abstract

**Background:**

Insecticide Treated Nets (ITNs) are an important tool for malaria control. ITNs are effective because they work on several parts of the mosquito feeding cycle, including both adult killing and repelling effects.

**Methods:**

Using an elaborated description of the classic feeding cycle model, simple formulas have been derived to describe how ITNs change mosquito behaviour and the intensity of malaria transmission, as summarized by vectorial capacity and EIR. The predicted changes are illustrated as a function of the frequency of ITN use for four different vector populations using parameter estimates from the literature.

**Results:**

The model demonstrates that ITNs simultaneously reduce mosquitoes' lifespans, lengthen the feeding cycle, and by discouraging human biting divert more bites onto non-human hosts. ITNs can substantially reduce vectorial capacity through small changes to all of these quantities. The total reductions in vectorial capacity differ, moreover, depending on baseline behavior in the absence of ITNs. Reductions in lifespan and vectorial capacity are strongest for vector species with high baseline survival. Anthropophilic and zoophilic species are affected differently by ITNs; the feeding cycle is lengthened more for anthrophilic species, and the proportion of bites that are diverted onto non-human hosts is higher for zoophilic species.

**Conclusion:**

This model suggests that the efficacy of ITNs should be measured as a total reduction in transmission intensity, and that the quantitative effects will differ by species and by transmission intensity. At very high rates of ITN use, ITNs can generate large reductions in transmission intensity that could provide very large reductions in transmission intensity, and effective malaria control in some areas, especially when used in combination with other control measures. At high EIR, ITNs will probably not substantially reduce the parasite rate, but when transmission intensity is low, reductions in vectorial capacity combine with reductions in the parasite rate to generate very large reductions in EIR.

## Background

Insecticide-treated nets (ITNs) are now regarded as standard tools for malaria control [1]. ITNs reduce malaria transmission by vectors that bite during the night; they work by reducing the intensity of malaria transmission, the average number of infectious bites received by a person over some time period, called the entomological inoculation rate (EIR). In one study, ITNs reduced EIR by a factor of 10 [2]. Previously published reports have demonstrated that the use of ITNs reduced mortality attributable to malaria by 30% in young children [3] and reduced severe, life-threatening malaria among children by 44% [4]. The benefits of ITNs may vary from place to place depending on both the baseline EIR and the total reduction in EIR [5,6].

ITNs are effective at reducing EIR because they simultaneously affect several different aspects of the mosquito feeding cycle. ITNs kill mosquitoes that land on treated nets, with mortality reaching up to 90% [7]. By increasing adult mortality, ITNs reduce transmission in at least four ways: a reduced probability that a mosquito will become infected, a reduced probability that an infected mosquito survives sporogony to become infectious, fewer expected bites by a vector after becoming infectious, and fewer eggs laid. ITNs also repel mosquitoes – in quantitative terms, mosquitoes are less likely to enter houses with ITNs and they leave sooner. The mean number of *Anopheles *mosquitoes per house was 77% lower in an area using ITNs than in a comparable one without ITNs [2]. Other effects have also been reported, including a shift to outdoor biting, a shift in time of biting, or diversion to feed on other blood meal sources [8].

The combined effects of ITNs, including both adult killing and repelling effects, are likely to be different, depending on the intrinsic biting preferences of the vector. Classical descriptions of malaria transmission are based on a quantitative description of the feeding cycle [9]. The mosquito feeding cycle involves host seeking, feeding, resting, oviposition-site seeking, and oviposition. Most formulas that describe malaria transmission by mosquitoes reduce this feeding cycle to a single term that describes the human feeding rate, *a*, where 1/*a *is the average interval between two successive human bites. The human feeding rate can be further broken down into two terms, the average time to complete one feeding cycle, 1/*f*, and the proportion of bites on humans, *Q*; the human feeding rate is *a *= *fQ*. The capacity of a single vector to transmit malaria is also affected by its lifespan and the duration of parasite sporogony; let *p *denote the probability of surviving one day and *n *the number of days required to complete sporogony. The total capacity for a vector population increases with the density of the vector population; let *m *be the population density of vectors divided by the population density of humans. One index of malaria transmission intensity is vectorial capacity, the number of infectious bites that would arise from all bites on a single infected human on a single day:C=mQ2f2pn−ln⁡p
 MathType@MTEF@5@5@+=feaafiart1ev1aaatCvAUfKttLearuWrP9MDH5MBPbIqV92AaeXatLxBI9gBaebbnrfifHhDYfgasaacH8akY=wiFfYdH8Gipec8Eeeu0xXdbba9frFj0=OqFfea0dXdd9vqai=hGuQ8kuc9pgc9s8qqaq=dirpe0xb9q8qiLsFr0=vr0=vr0dc8meaabaqaciaacaGaaeqabaqabeGadaaakeaacqWGdbWqcqGH9aqpdaWcaaqaaiabd2gaTjabdgfarnaaCaaaleqabaGaeGOmaidaaOGaemOzay2aaWbaaSqabeaacqaIYaGmaaGccqWGWbaCdaahaaWcbeqaaiabd6gaUbaaaOqaaiabgkHiTiGbcYgaSjabc6gaUjabdchaWbaaaaa@3D27@[10]. Vectorial capacity is closely related to other indices of malaria transmission, including EIR and R_0 _[11,12]. Here, the differences in bed-net effects are assessed using a mathematical model for the change in transmission intensity produced by ITN use, based on a detailed quantitative description of the mosquito feeding cycle, extending previous work on mosquitoes and malaria transmission [9,13,14]. The study aims to improve understanding of how host-feeding by mosquitoes would be influenced by the use of ITNs, and to show how ITNs can have similar reductions in EIR for different reasons, despite innate differences in mosquito ecology.

## Methods

**Figure 1:**
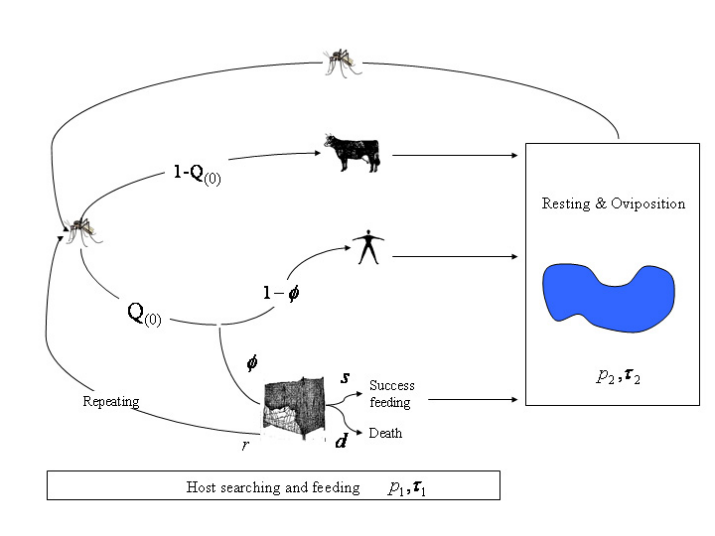
Mosquito feeding cycle. The cycle is divided into three parts a) the host searching process: mosquitoes complete the cycle within *τ*_1 _days and survive this process with a probability *p*_1_. They may visit animals or humans at a probability *H*(= *Q*_(0)_). They may encounter humans protected by ITNs at a probability *φ*. If so, mosquitoes may successfully bite at a probability *s*, die after landing on the net at a probability *d *or repeat the host searching cycle at a probability *r *until they successfully feed. b) Once mosquitoes have successfully fed they rest during *τ*_2 _days and survive at a probability *p*_2_.

Here, the mosquito feeding cycle is described by 2 stages: host-seeking through to successful feeding, and resting through to oviposition (Figure 1). To understand ITNs, all the events that are changed by ITNs when a mosquito bites or attempts to bite a host are examined in details (Figure 1). First, during the host-seeking period, a proportion *Q*(0) of mosquitoes find a human, and 1-*Q*(0) find some other vertebrate host. With ITN use, a fraction *φ *of the humans are protected by ITNs. If a mosquito finds a protected human, one of three things can happen: a) the mosquito successfully feeds, regardless of the net, with probability *s*. Successful feeding on humans protected by ITNs may occur if the net has holes, if the net is not properly deployed, or if the human is unprotected and bitten for short stretches during the night b) the mosquito lands on the net to bite a human, but dies from contact with the insecticide, with probability *d *c) the mosquito leaves the vicinity to search for another host, effectively restarting the host-seeking cycle, with probability *r *= 1 - *s *- *d*. In summary, at the end of one biting attempt, some mosquitoes have successfully fed on a non-human host, some have fed on a human, some have died from the insecticide in the net, and some have been frustrated and begin again.

Since the study is interested in understanding how ITNs affect malaria transmission changes to vectorial capacity are assessed in a controlled population relative to a baseline population without control. Without ITNs, host-seeking takes *τ*_1_(0) days and mosquitoes survive this process with a probability *p*_1_(0). Following a successful blood meal, mosquitoes rest, find larval habitat, and oviposit. This process lasts *τ*_2 _days and the mosquito survives with probability *p*_2 _regardless of species biting preferences. This elaborated description is related to classic formulations: the time to complete one feeding cycle with no ITN use is 1/*f*(0) = *τ*_1_(0) + *τ*_2_, and the daily probability of surviving one day is *p*(0) = [*p*_1_(0) *p*_2_]^*f*(0)^. The proportion of blood meals that are taken on a human is also affected by ITNs; let *Q*(0) denote the proportion of blood meals taken on a human in the absence of ITNs, i.e. the human visiting rate. Functions were elaborated to describe vectorial capacity in a population where a fraction of humans, *φ*, are protected by ITNs; in other words, what are *f*(*φ*), *p*(*φ*), and *Q*(*φ*)?

A surviving, diverted mosquito can successfully feed after several attempts. To derive the following formulas, we assume that they may repeat the attempt as many times as necessary to complete their feeding cycle. A mosquito successfully feeds by finding a non-human host, by finding an unprotected human host, or by successfully feeding on a protected human host. Altogether, during a single attempt, a surviving mosquito succeeds with probability *W *= 1 - *Q*(0) + *Q*(0) (1-*φ*) + *Q*(0) *φs *= 1 - *Q*(0) *φ *(1-*s*). A mosquito resets and begins a new search with probability *Z *= *Q*(0) *φr*, assuming that the mosquito survived the ordinary hazards of feeding and seeking, that it survived the ITN, and that it failed to feed successfully.

First, a formula was elaborated for the expected delay. By assumptions, after a failed first attempt, the mosquito starts over, with probability Z, so the expected time to complete the life-cycle is given by:

*τ*_1_(*φ*) = *τ*_1_(0) + *Zτ*_1_(*φ*) = *τ*_1_(0)/(1-*Z*);     *(Equation 1)*

Intuitively, this expression says that the expected time to feed in a population where ITNs are used is the baseline time multiplied by the number of attempts required to complete a feeding cycle, 1/(1-Z). Thus, the time to complete one feeding cycle is

1/*f*(*φ*) = *τ*_1_(0)/(1-*Z*) + *τ*_2_;     *(Equation 2)*

Second, a formula was elaborated for the expected probability that a mosquito survives through completion of feeding. At each attempt, mosquitoes may die, succeed, or fail and try again. Thus the probability of surviving through a complete feeding cycle is the probability of surviving and succeeding the first attempt, plus the probability of surviving the first attempt and succeeding the second attempt if mosquitoes failed the first attempt, and so on. Importantly, we assume that the baseline hazards apply each time a mosquito attempts to feed:

*p*_1_(*φ*) = *p*_1_(0) [*W *+ *Z p*_1_(*φ*)] = *p*_1_(0)*W*/[1 - *Z p*_1_(0)];     *(Equation 3)*

Thus, the probability of surviving one day is given by:

*p*(*φ*) = [*p*_1_(0) *p*_2_*W*/(1-*Z p*_1_(0)]^*f*(*φ*)^;     *(Equation 4)*.

Finally, with ITN use, a larger fraction of bites is taken on non-human hosts. Overall, the probability that a feeding attempt ends with a blood meal on a human host is:

*q*(*φ*) = *p*_1_(0) [*Q*(0)(1 - *φ *+ *φ s*) + *Z q*(*φ*)]

= *p*_1_(0) *Q*(0)(1 - *φ *+ *φ s*)/[1-*Z p*_1_(0)]     *(Equation 5)*

The proportion of blood meals taken on a human host is:

*Q*(*φ*) = *q*(*φ*)/*p*_1_(*φ*) = *Q*(0)(1 - *φ *+ *φ s*)/*W*.     *(Equation 6)*

Thus, three formulas have been derived (Equation 2, 4 and 6) relating the probability of surviving the feeding cycle, the average length of a feeding cycle, and the proportion of bites taken on a human to the analogous baseline rates in the absence of ITNs as a function of ITN coverage, φ. The functions rely on only two additional parameters: the probability that the average mosquito dies, *d*, or that it feeds successfully, *s*, during a single feeding attempt.

Finally, ITNs can have other effects on vector population density. By killing adult mosquitoes, the total rate of oviposition goes down in the surrounding area. More importantly, there are direct reductions in adult vector density. The rate at which mosquitoes emerge, per-human, per day, is a constant *λ*; it follows that mosquito density is reduced slightly because of the higher death rates, to *m*(*φ*) = *λ*/-ln *p*(*φ*) Thus, the total effect of ITNs on vectorial capacity can be rewritten as a function of each one of these separate effects:

C(φ)=m(φ)Q(φ)2f(φ)2p(φ)n−ln⁡p(φ)=λQ(φ)2f(φ)2p(φ)n(−ln⁡p(φ))2     (Equation 7)
 MathType@MTEF@5@5@+=feaafiart1ev1aaatCvAUfKttLearuWrP9MDH5MBPbIqV92AaeXatLxBI9gBaebbnrfifHhDYfgasaacH8akY=wiFfYdH8Gipec8Eeeu0xXdbba9frFj0=OqFfea0dXdd9vqai=hGuQ8kuc9pgc9s8qqaq=dirpe0xb9q8qiLsFr0=vr0=vr0dc8meaabaqaciaacaGaaeqabaqabeGadaaakeaacqWGdbWqcqGGOaakiiGacqWFgpGzcqGGPaqkcqGH9aqpdaWcaaqaaiabd2gaTjabcIcaOiab=z8aMjabcMcaPiabdgfarjabcIcaOiab=z8aMjabcMcaPmaaCaaaleqabaGaeGOmaidaaOGaemOzayMaeiikaGIae8NXdyMaeiykaKYaaWbaaSqabeaacqaIYaGmaaGccqWGWbaCcqGGOaakcqWFgpGzcqGGPaqkdaahaaWcbeqaaiabd6gaUbaaaOqaaiabgkHiTiGbcYgaSjabc6gaUjabdchaWjabcIcaOiab=z8aMjabcMcaPaaacqGH9aqpcqWF7oaBdaWcaaqaaiabdgfarjabcIcaOiab=z8aMjabcMcaPmaaCaaaleqabaGaeGOmaidaaOGaemOzayMaeiikaGIae8NXdyMaeiykaKYaaWbaaSqabeaacqaIYaGmaaGccqWGWbaCcqGGOaakcqWFgpGzcqGGPaqkdaahaaWcbeqaaiabd6gaUbaaaOqaaiabcIcaOiabgkHiTiGbcYgaSjabc6gaUjabdchaWjabcIcaOiab=z8aMjabcMcaPiabcMcaPmaaCaaaleqabaGaeGOmaidaaaaakiaaxMaacaWLjaacbiGae4hkaGIaemyrauKaemyCaeNaemyDauNaemyyaeMaemiDaqNaemyAaKMaem4Ba8MaemOBa4MaeeiiaaIae43naCJae4xkaKcaaa@8148@

Using the classical assumptions, changes in vectorial capacity are related to changes in the entomological inoculation rate by the formula:

EIR (φ) = b C (φ) X(φ)/[1 + Q(φ) f(φ) c X(φ)/-ln p(φ) ]     *(Equation 8)*

where X(φ) denotes the parasite rate, the fraction of humans that carry parasites, c is the infectivity, the fraction of bites on infected humans that would infect a mosquito (i.e the fraction of parasitemic humans that transmit gametocytes to mosquitoes, per bite) and b is the probability a human gets infected from mosquitoes bites.

Sample parameters describing the baseline feeding cycle process result from epidemiological surveys [15-17] and are listed in Table 1.

**Table 1:** Symbols, definitions and values (bounds) for input and model parameters (* parameters included in the multivariate sensitivity analysis)

Parameter	Name	Definition	*Anopheles gambiae *s.l. (N)	*Anopheles arabiensis*	*Anopheles gambiae *s.l. (T)	*Anopheles punctulatus*	References
*p*	Daily survival rate	Probability that a mosquito survive throughout the cycle and is not killed by host defensiveness or predation	0.90	0.94	0.83	0.86	[16]
*H*	Human visiting rate	Probability that a mosquito visit a human to feed on	0.90	0.75	0.95	0.72	[16]
*φ*	ITN coverage	Proportion of humans sleeping under ITNs	0–1	0–1	0–1	0–1	This paper
*n*	Average incubation period in days	Delay before mosquito become infectious after being infected	10.3	11.6	10.7	8.3	[16]
*s*	Successful protected human biting*	Probability that a mosquito successfully bites after finding a human under ITNs	0.1 **(0.1–0.6)**	0.1	0.1	0.1	[15]
*d*	Insecticide mortality rate*	Probability that a mosquito is killed by insecticide after finding a human under ITNs	0.3 **(0.05–0.3)**	0.3	0.3	0.3	[15,17]
*r*	Cycle repeating rate*	Probability that the mosquito looks for another host after finding a human under ITNs	0.6 **(0.1–0.85)**	0.6	0.6	0.6	[17]

### ITNs impact on vectors behavior and on malarial key parameters

These formulas were applied to three mosquito species in different regions: anthropophilic species, i.e. *Anopheles gambiae *s.l. in Nigeria (N) and in Tanzania (T) and more zoophilic species, i.e. *Anopheles arabiensis *in Nigeria and *Anopheles punctulatus *in Papua New Guinea. The study aims to gain a better quantitative understanding of how ITN coverages ranging from 0% to 100% influence parameters that describe vector ecology and malaria, that is, mosquito lifespan, duration of the feeding cycle, proportion of bites taken on human hosts, changes in vectorial capacities and in EIR.

### ITNs loss of efficacy over time

The efficacy of ITNs and their impact on the five previously described parameters were assessed by modifying characteristics of ITN effects over time; the effect of insecticide progressively fades, so the feeding success rate linearly was assumed to increase from 0.1 to 0.6 (at which point the insecticide effect completely disappeared) and the death rate dropped from 0.3 to 0.05. This analysis was carried out for the anthropophilic species *A. gambiae *s.l. in Nigeria.

### Uncertainty on ITNs characteristics and impact on malaria transmission

A multivariable sensitivity analysis was also performed on two parameters that describe ITNs, that is the feeding success rate, *s *and death rate when landing on ITNs, *d*. These two parameters were sampled using a Latin Hypercube Sampling scheme [18] from uniform continuous distribution with bounds described in Table 1. The effect of variation in these parameters values on the intensity of malarial transmission through vectorial capacity was explored. This analysis was carried out for the anthropophilic species *A. gambiae *s.l. in Nigeria (N).

## Results

### How do ITNs impact vector behavior and malaria transmission?

**Figure 2:**
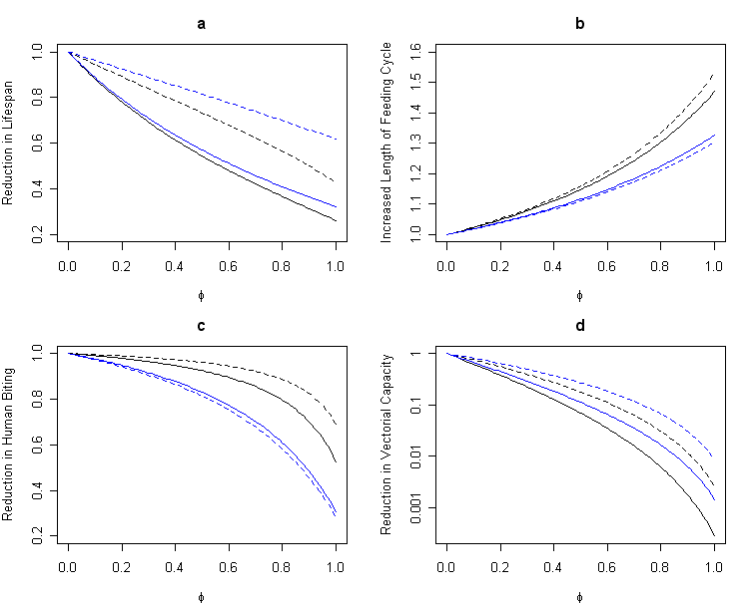
Effect of bednet coverage on vectors behavior and on malarial key parameters. a) Proportional reduction in mosquito life span, b) Proportional increase in duration of the feeding cycle, c) Proportional reduction in proportion of bites on humans (*Q*), and d) Proportional reduction in the logarithm of vectorial capacity as a function of ITN coverage,*φ*, for different species and different geographical locations. Anthropophilic species: *Anopheles gambiae *s.l. (N) in Nigeria (solid black line) and *Anopheles gambiae *(T) in Tanzania (dashed black line). Zoophilic species: *Anopheles arabiensis *in Nigeria (solid blue line) and *Anopheles punctulatus *in Papua New Guinea (dashed blue line).

ITNs act by reducing the mosquito survival rate throughout the feeding cycle. Mean mosquito lifespan falls rapidly as ITN coverage increases due to extra mortality when mosquitoes land on a net. For instance, the mean lifespan of *A. gambiae *s.l. (N) decreases to approximately one-fourth of the baseline (from 9.5 days with no ITNs to 2.5 days with full ITN coverage, Figure 2a). The greatest decreases are observed when mosquito survival rates throughout the cycle are initially high regardless of species preference (anthropophilic or zoophilic). Thus, for the zoophilic species *A. arabiensis*, with an estimated daily probability of surviving a feeding cycle of 0.94, full ITN coverage cuts mosquito lifespan to a third of the baseline (from 16.2 days to 5.2 days) whereas for the more zoophilic species *A. punctulatus*, with a daily survival rate of 0.86, the lifespan is reduced to about two-thirds of the baseline (from 6.6 to 4.1 days, Figure 2a). Similar patterns are observed with the two anthropophilic species *A. gambiae *s.l. (N) and (T). In addition to reducing the daily survival rate, ITN use increases the duration of the feeding cycle; the effects are larger, depending on the baseline fraction of bites taken on a non-human. Anthropophilic species require more time to find a host when ITN coverage increases. The feeding cycle for *A. gambiae *s.l. (N) is lengthened by about 50%, from 2 days to 2.9 days. By contrast, zoophilic mosquitoes are diverted to other non-human vertebrate hosts, leading to lesser increases in the feeding cycle duration. The feeding cycle for *A. arabiensis *is lengthened by about one-third, from 3 to 4 days (Figure 2b). Finally, the proportion of bites taken on a human declines more for a zoophilic species than an anthrophilic species. For instance, with full ITN coverage, approximately one-fifth of *A. punctulatus *bites are taken on a human (a 70% decrease) versus two-thirds with *A. gambiae *s.l. (T) (a 30% decrease, Figure 2c).

One way of summarizing the combined effects of ITNs is to estimate the reduction in the number of feeding cycles a mosquito would complete over its lifespan. For example, with full ITN coverage, *A. gambiae *s.l. (N) completes approximately one-sixth as many feeding cycles as the baseline (dropping from 4.7 to 0.8 cycles). The same trend is observed with the other species, but not to the same extent; for example *A. punctulatus *would complete about half as many life-cycles (decreasing from 1.8 to 0.85). Thus, the period during which a mosquito is infectious and able to transmit the infection would also be greatly reduced.

The relationship between vectorial capacity and ITN use summarizes the total effects of all these relationships. As ITN coverage increases, vectorial capacity exponentially decreases; with complete coverage, ITNs can reduce vectorial capacity by a factor of 50 to 100 or more (Figure 2d). Thus similar trends in vectorial capacity are observed regardless of species biting preference or survival rate. A 50% ITN coverage leads to a reduction in vectorial capacity, ranging from 72.1% for *A. punctulatus *to 92.7% for *A. gambiae *s.l. (N) (Figure 2d). Species biting preference, anthropophilic or zoophilic, does not seem to influence the total reduction in vectorial capacity values, as the largest reductions in vectorial capacity values are observed for species with the highest baseline daily survival rates. For instance, similar vectorial capacities are observed for zoophilic species such as *A. arabiensis *(*C *= 8.63 when no ITNs are used) and anthropophilic species such as *A. gambiae *s.l. (N) (*C *= 5.37), which exhibit comparable baseline survival rates (*An. gambiae *s.l. (N) *p*_*cycle *_= 0.81 and *An. arabiensis p*_*cycle *_= 0.83). Similar patterns are observed for low-cycle-survival-rate species – *A. gambiae *s.l. (T) (C = 0.49 when no ITNs are used) and *A. punctulatus *(C = 0.48).

**Figure 3:**
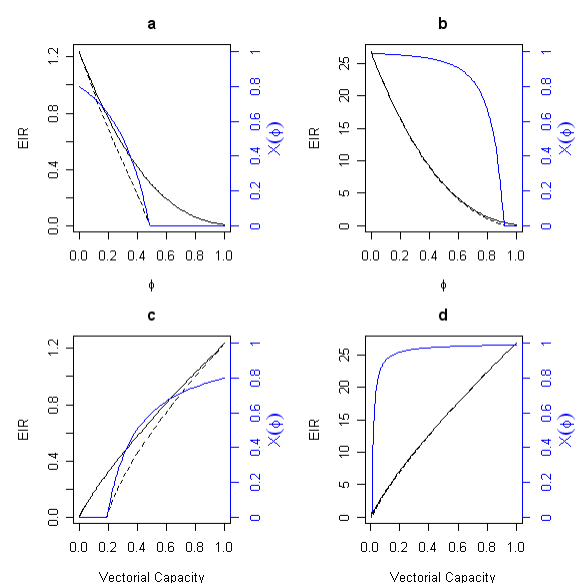
Effect of bednet coverage in EIR values for various human prevalence levels. EIR as a function of φ at low (a) and high (b) transmission intensities and as a function of vectorial capacity at low (c) and high (d) transmission intensities for *Anopheles gambiae *in Tanzania. The solid line gives the immediate reduction in EIR due to the reduction in vectorial capacity. The blue line shows the reduction in the prevalence of malaria infection (X(φ), computed with the classical model for b = 0.8, 1/r = 200 days, and c = 0.8 [11,13,19]. The dashed line shows the eventual reduction in EIR after the reductions in malaria prevalence take effect (probably after a few years)

EIR values follow a similar exponential decrease as ITN coverage increases, but the effects differ at low and high baseline transmission intensities. The prevalence of malaria parasitemia in humans following effective ITN deployment is more likely to decline when baseline transmission intensity is low (i.e. annual EIR below 1), than when EIR is high [19]. Changes to EIR include changes in the fraction of infected humans – at low EIR, bed-nets will reduce human prevalence which will generate further reductions in EIR (Figure 3), albeit after a delay.

### How effective are ITNs after the insecticide weakens?

**Figure 4:**
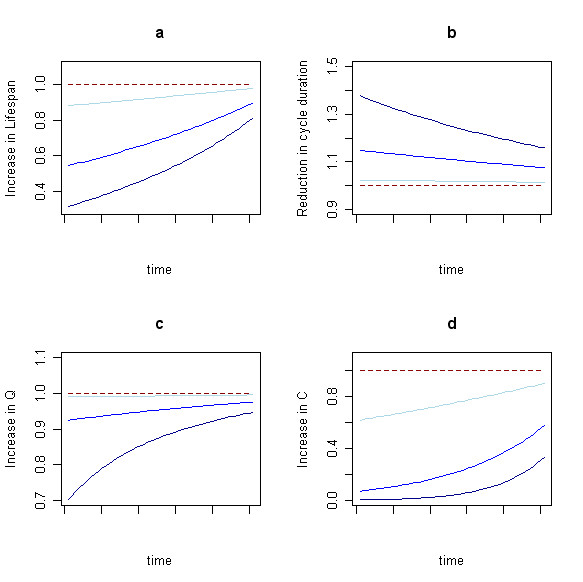
Effect of bednet loss of efficacy over time a) Increase in mosquito life span, b) Reduction in duration of the feeding cycle, and c) Increase in proportion of bites on humans (*Q*), and d) Increase in vectorial capacity, as a function of ITN efficiency over time (successful feeding rate *s *increases linearly from 0.1 to 0.6 over time and death rate *d *when landing on ITNs decreases from 0.3 to 0.05 over time) for the anthropophilic species *Anopheles gambiae *s.l. (N) in Nigeria for 3 different values of ITN coverage *φ*: low ITN coverage (*φ *= 10%; light blue), medium ITN coverage (*φ *= 50%; blue line), high ITN coverage (*φ *= 90%; dark blue line). The red dashed line corresponds to the standard reference when no ITNs are used.

As insecticide effects weaken, mosquito lifespan increases compared to the full-efficacy control levels (Figure 4a), the duration of the feeding cycle is shortened (Figure 4b), and fewer mosquitoes are diverted to bite non-human hosts (Figure 4c). As a result, vectorial capacity (Figure 4d) becomes more similar to pre-control baseline levels without reaching them. Whereas with an initial low ITN coverage (10%) the lost efficacy is slight, for an initial high ITN coverage (90%), those effects are relatively large and clearly identified from lower ITN coverage, particularly regarding the proportion of bites on humans (Figure 4c; with a 90% ITN coverage 85% of the bites are taken on humans when the insecticide is no longer effective, a 35% increase compared to full insecticide efficiency) and the increase in vectorial capacity (Figure 4d: values are more than 100 times greater with a 90% ITN coverage after insecticide effects fade out, but only 1.5 times greater with an initial 10% ITN coverage).

### How do ITN characteristics influence malaria transmission?

**Figure 5:**
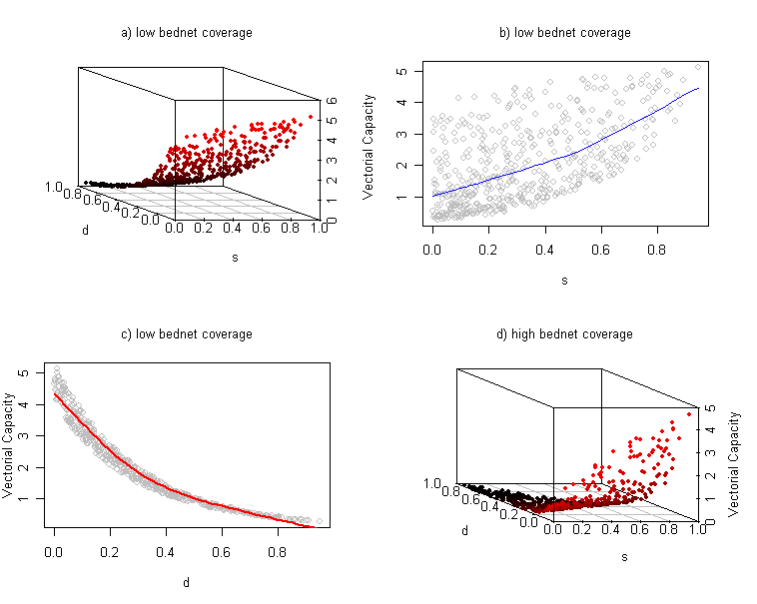
Multivariable sensitivity analysis for ITN parameters: successful feeding rate *s *and death rate *d *when landing on ITNs for the anthropophilic species *Anopheles gambiae *s.l. (N) in Nigeria. a) Impact of the two parameters on Vectorial Capacity under low ITN coverage (10%). b) Impact of the successful feeding rate through ITNs on vectorial capacity under low ITN coverage (10%); the blue line represents non- parametric local polynomial regression fitting. c) Impact of the death rate s on vectorial capacity under low ITN coverage (10%); the red line represents non-parametric local polynomial regression fitting. d) Impact of the two parameters on Vectorial Capacity under high ITN coverage (90%).

The results of the multivariable sensitivity analysis on two ITN parameters demonstrated that the most influential parameter on vectorial capacity (under low ITN coverage (10%)) is the death rate due to landing on a treated ITN, *d*, followed by the feeding success rate despite the presence of an ITN, *s *(Figure 5b, c). Greater variation in vectorial capacities is observed when vectors have similar feeding success rates (Figure 5b) than when they have similar death rates (Figure 5c). Under higher ITN coverage, vectorial capacity exponentially increases above a certain threshold estimated to be close to assumed values for a non-treated net (s = 0.6 and d = 0.05; Figure 5d); whereas with low ITN coverage, vectorial capacity increases linearly as feeding success rate increases and death rate decreases (Figure 5a).

## Discussion

Using an elaborated description of the classic feeding cycle model, simple formulas have been derived to describe how ITNs change the intensity of malaria transmission, as summarized by the vectorial capacity. ITNs accomplish these changes by reducing daily survival (and by extension, mosquito density), increasing the duration of the mosquito feeding cycle, and decreasing the proportion of blood meals taken on a human host. ITNs affect all these aspects of transmission for all vectors, but the dominant effects differ for anthropophilic and zoophilic vectors. ITNs reduce vectorial capacity mainly through reductions in vector lifespan; the effects are largest for species with high baseline survival, but these effects are enhanced by lengthening the feeding cycle and diverting bites onto non-human hosts. The model also predicts that ITNs delay feeding more for anthropophilic vectors. For zoophilic vectors, ITNs increase the proportion of bites that are taken on non-human hosts. Despite these differences, the total reduction in transmission is similar, and is strongly affected by the baseline mosquito ecology. As a result, the risk of malaria would be reduced more and the vectorial capacity would decrease by more than 90% in any species, regardless of host biting preferences.

The risk of an infection is related to changes in EIR, but such changes will differ enormously depending on the absolute value of EIR at the baseline, not just the proportional reductions in vectorial capacity. Baseline EIR and vectorial capacity can greatly vary because of mosquito density, a quantity that is related to mosquito larval ecology. While ITNs can reduce the number of eggs laid by adult mosquitoes, this may not reduce the number of emerging adults, especially if larval populations are regulated in a density dependent way by other factors, especially competition for resources and predation.

When baseline EIR is high, a large proportional reduction in vectorial capacity (i.e. by a factor of 100) may simply reduce EIR from 700 to 7, but an annual EIR of 7 still represents a high level of risk. Reductions from extremely high EIR with ITNs can produce short-term reductions in morbidity and mortality that reflect residual levels of immunity acquired under baseline transmission. The long-term effects, all else equal, are expected to adjust such that morbidity and mortality in the controlled populations would be expected to resemble other places with that baseline EIR. The real unanswered question is whether the total burden of malaria is importantly different in populations with high baseline EIR. How different is the total burden of malaria in a population where annual EIR is constantly 7 compared to one where it is 70 or 700 [5]?

These models also suggest that complete ITN use can generate reductions in transmission intensity [20] that are high enough to locally eliminate malaria from some areas where the intensity of transmission is low or moderate (i.e. by a factor of a hundred or more) if all transmission is by night-biting mosquitoes and if ITN use is high (i.e. in excess of 50%). At low EIR, large proportional changes in vectorial capacity might lead to significant reductions in parasite rate, and consequently the proportion of mosquitoes that become infected per bite. The added reductions in human prevalence might happen after a delay, but the combined effects of reductions in vectorial capacity and reductions in the parasite rate will produce even larger changes in EIR when baseline EIR is low.

The effects of ITNs might have different important effects in areas with perennial transmission compared with acutely seasonal transmission, and this raises important questions about the timing of insecticide treatments and public health messages to promote ITN use [21]. Should ITNs be emphasized and treated during times of high or low transmission? In some places, ITN use during the dry season combined with mass drug administration could be used to knock out the cryptic human infections that spark transmission during the following season [22].

Here, ITN use is considered in terms of the proportion of mosquito-human encounters with an ITN-protected human; this is the relevant quantity for malaria transmission, but it is obviously a different definition than ITN coverage, the proportion of households that own a ITN. Achieving 100% ITN coverage may be unrealistic. In the Gambia, where the use of ITNs is widely accepted, ITN coverage evaluated during the National Insecticide Impregnated Bed-net (ITN) Programme ranged from 46.4% to 78.6% in five different villages [23]. Furthermore, ITNs may become inefficient if not re-impregnated regularly, and the rate of re-treatment may vary from 7% to 62% after introduction of cost-retrieval [24]. Thus, ITN evaluation through mathematical models should take into account realistic use of ITNs, as is done here; mosquitoes are allowed to bite through inefficient nets.

The cycling model does not take into account heterogeneous distributions of mosquitoes or hosts [27], but it has been shown that mosquitoes' demographic characteristics influence malaria risk [14]. For instance, villages close to larval habitats are at higher risk of malaria infection [25]. Nevertheless, the model results are in agreement with epidemiological studies. In this model, decreases in daily survival rate range from 9 to 26%, similar to the 10 to 40% decreases observed in field surveys [26]. To mimic real contexts, the input and model parameters used are developed from studies carried out in Africa, in Papua New Guinea [16,17] or in Latin America [15]. Finally, the model provides simple formulae for mosquito survival rate, duration of the feeding cycle and proportion of bites taken on humans.

This approach provides a framework for predicting ITN efficacy based on mosquito characteristics and behavioural response in relation to ITN use. Such methods provide tools for planning interventions and evaluating other measures such as the use of untreated ITNs [28]. The results of the model suggest that malaria risk would be greatly reduced by medium ITN coverage regardless of species biting preference, thereby providing important observations with clear public health implications to achieve sufficient protection and control of malaria.

## Authors' contributions

ALM refined the model, performed the sensitivity analysis, carried out the simulations and wrote the manuscript. ST, AP and AH designed the original model and reviewed the manuscript. FEMK actively participated to the follow-up of the study and helped to draft the manuscript. AF helped to draft the manuscript. DLS designed and supervised the study, built the model and helped to write the manuscript.
